# Prediction Models of Infective Endocarditis Usable Ahead of Performing Blood Cultures: A Narrative Review

**DOI:** 10.7759/cureus.78754

**Published:** 2025-02-08

**Authors:** Shun Yamashita, Masaki Tago, Kota Minami, Naoko E Katsuki, Yasutomo Oda, Shu-ichi Yamashita

**Affiliations:** 1 Department of General Medicine, Saga University Hospital, Saga, JPN; 2 Education and Research Center for Community Medicine, Faculty of Medicine, Saga University, Saga, JPN; 3 Center for Graduate Medical Education Development and Research, Saga University Hospital, Saga, JPN; 4 Department of Internal Medicine, Heiwadai Hospital, Miyazaki, JPN

**Keywords:** area under the curve (auc), generalizability, infective endocarditis, narrative review, prediction model, strength

## Abstract

Infective endocarditis (IE) often presents as a fever of unknown origin due to its extremely diverse clinical presentations, requiring diverse advanced medical equipment and tests to make a correct diagnosis. Whether a physician can suspect IE in a clinical setting is dependent on the physician’s knowledge and experience. If IE is not suspected, antibiotics are administered without obtaining blood cultures, complicating the clinical course and prognosis. To avoid delayed diagnosis or entering the maze of diagnostic difficulties of IE cases, a prediction model to deduce IE likelihood can be used at an early stage after a patient’s arrival at the hospital before blood culture examinations would be invaluable. In this study, we aimed to review the literature on such prediction models for IE diagnosis in existence, discussing their strengths and limitations. A narrative review was conducted by two researchers using PubMed. Comprehensive searches included the index terms "infective endocarditis" or "infectious endocarditis", coupled with "prediction model" or "prediction rule" or "predictive model". Five articles reporting one of the three prediction models were identified. The first model, developed for intravenous drug users (IDUs) admitted to the emergency departments of two to three hospitals showed a good area under the curve (AUC) of 0.8; however, the small sample size and overfitting of the model were a limit. The second model for inpatients in all departments of four hospitals showed an AUC of 0.783 with a shrinkage coefficient of 0.963, indicating high generalizability. Moreover, it featured the highest ease of use because it consisted of only five factors readily available in any hospital. The third model, developed for inpatients admitted to an emergency department at a single center, consisted of 12 factors and achieved the highest AUC (0.881). All models demonstrated fair to good AUC. The second model excelled in generalizability and ease of use, while the third model was superior in performance. To further improve the accuracy of each IE prediction, further high-level evidence studies, such as randomized controlled trials in multiple facilities, are mandatory.

## Introduction and background

Infective endocarditis (IE) presents with extremely diverse clinical symptoms including fever, general malaise, dyspnea, arthralgia, back pain, abdominal pain, altered consciousness, and limb paralysis [1]. It is a known cause of fever of unknown origin, accounting for approximately 5% of all diseases that cause such fever [1,2]. Despite advancements in diagnostic tools and antibiotics, the mortality rate remains high at 13-18% [1,3]. Until 2022, IE was diagnosed using the modified Duke criteria, which require various advanced medical modalities, such as multiple blood cultures, transthoracic or transesophageal echocardiography, computed tomography, and magnetic reasoning imaging [4]. However, the modified Duke criteria had the drawback of a low sensitivity of 52% for detecting IE in patients with prosthetic valves [5]. In 2023, the Duke-ISCVID IE criteria were proposed as updated diagnostic criteria for IE, offering enhanced diagnostic capabilities [6]. The Duke-ISCVID IE criteria rely more heavily on advanced diagnostic tools, such as 18F-fluorodeoxyglucose positron emission tomography and cardiac computed tomography, than the modified Duke criteria. Therefore, hospitals and clinics lacking such medical equipment may fail to make definitive diagnoses, which can cause difficulty in determining the need for referrals to higher-level medical institutions for further evaluation. Even in well-equipped hospitals, it is impractical to perform all diagnostic examinations required for IE in all cases of fever of unknown origin. Therefore, diagnosing IE in clinical practice often depends on the knowledge and experience of the physician. Due to its diverse manifestation, it is not feasible for all physicians to appropriately suspect IE when seeing patients with fever because they may visit various medical departments [3]. Failure to suspect IE can lead to the premature administration of inappropriate antibiotics without obtaining blood culture samples, resulting in the recurrence of IE after antibiotic administration is prematurely completed [7,8]. In such a case, previously administered antibiotics could hinder the detection of the causative bacteria through subsequent blood culture examinations [8,9]. Moreover, it can worsen the prognosis [8,9]. A prediction model to help physicians suspect IE in all clinical settings is invaluable in avoiding diagnosis delays or diagnostic difficulties of IE cases.

Several prediction models for IE have been reported with a sensitivity of 83-100% and a specificity of 15-91%; however, they are only applicable after blood culture examinations detect the representative bacteria of IE, such as *Staphylococcus aureus*, *Enterococcus*, and *Streptococcus *species [10-14]. Physicians may struggle to avoid prematurely administering antibiotics due to the relatively long wait time for blood culture results required by such models before suspecting IE. Therefore, it is crucial to develop diagnostic prediction models for IE that do not depend on blood culture results and can be immediately used after evaluating the patient. In this study, we reviewed the literature on such prediction models for IE, discussed the strengths and limitations of each model, and explored the desirability of developing models with both high performance and generalizability.

## Review

Methodology

Search Strategy

To identify studies that developed diagnostic prediction models for IE applicable shortly after a patient’s arrival and before performing blood cultures, a comprehensive search was conducted by two researchers. The search terms included a combination of "infective endocarditis" or "infectious endocarditis" and "prediction model", "prediction rule", or “predictive model”. These terms were used as the index terms in a comprehensive search with PubMed (Appendix 1). Only articles published in English from January 1781 to October 2024 were included. Each researcher independently determined which articles should be included in the present review and then discussed whether they could reach an agreement. Articles were registered as eligible if both researchers agreed; in case of disagreement, the researchers checked the inclusion and exclusion criteria together, re-selecting articles to be included in this review. When an agreement was reached, articles were registered as eligible. In case of repeated disagreement, the step was repeated. Here, the inclusion and exclusion criteria were checked only once (agreement rate: 98.3%, Cohen's kappa 0.58).

Inclusion and Exclusion Criteria

 Research articles were included in this review if they met all the following criteria: (1) original research involving human subjects, (2) focused on prediction models for the diagnosis of IE, and (3) without using the blood culture results. Review articles, case reports, and animal studies were excluded.

Results

**Figure 1:**
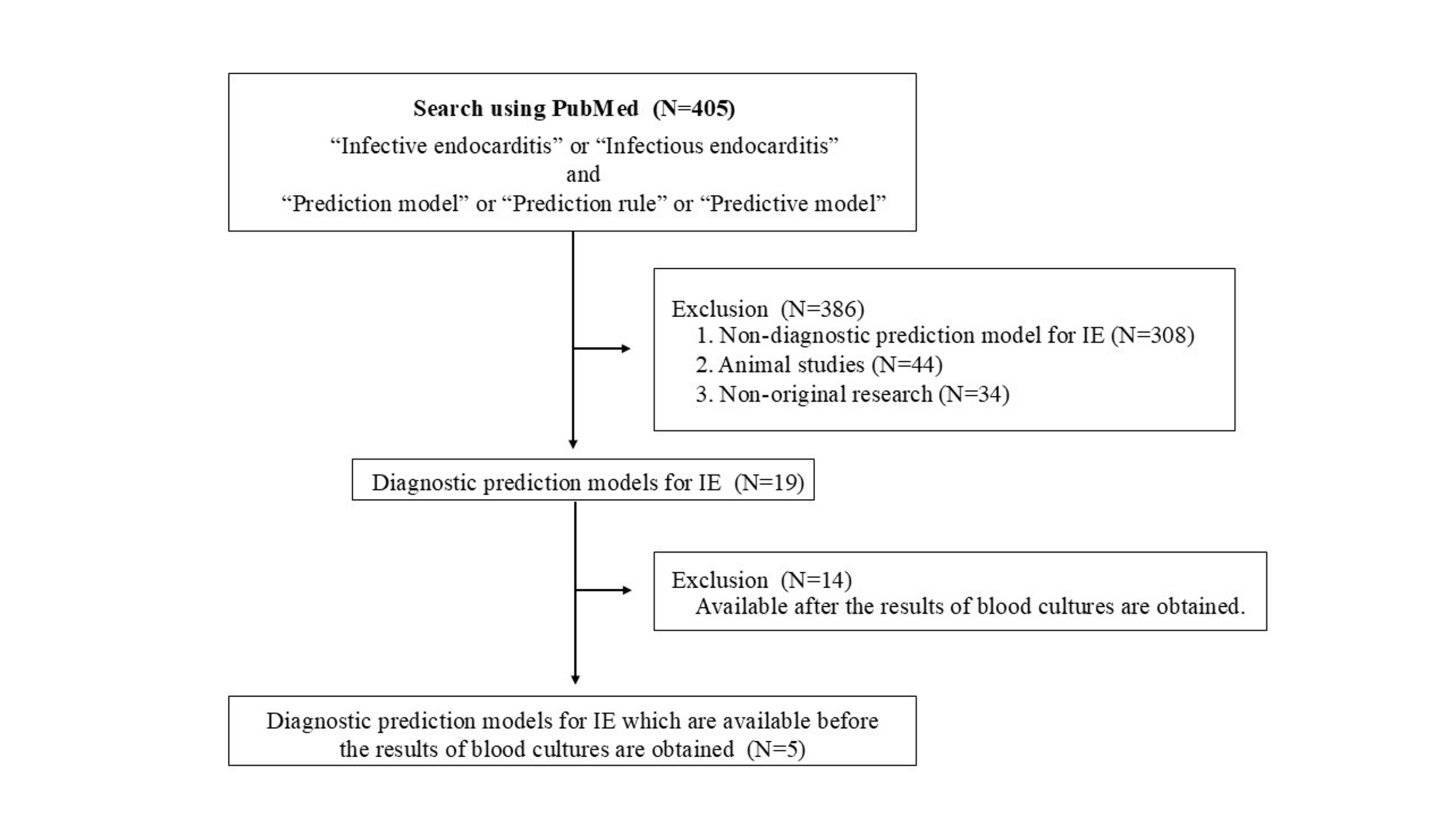
Review protocols of the present study Two researchers conducted a PubMed search using the index terms "infective endocarditis" or "infectious endocarditis" combined with "prediction model", "prediction rule", or "predictive model". A total of 405 articles were identified in PubMed. Among these, 308 articles that were not diagnostic prediction models for IE, 44 animal studies, and 34 non-original research articles were excluded. From the remaining 19 articles, 14 were excluded because their models required blood culture results. Ultimately, five articles were included in this review.

Using the index terms described above, 405 articles were identified in PubMed. Among these, 308 articles that were not diagnostic prediction models for IE, 44 animal studies, and 34 non-original research articles were excluded. From the remaining 19 articles, 14 were excluded as their models required blood culture results. Finally, five articles describing three diagnostic prediction models were included in this review (Figure 1). Summaries and formulas for each model are presented in Table 1 and Appendix 2, respectively.

**Table 1:** Difference among IE prediction models usable before performing blood cultures IE-IDU model: prediction model for infective endocarditis in injection drug users, CREED score: Clinical Rule for Infective Endocarditis in the Emergency Department, modified DC: modified Duke criteria Categorical data are expressed as n(%). §Patient’s age is are expressed as median (interquartile range) except the article written by Chung-Esaki H et al., which is expressed as mean ± standard deviation.

	IE-IDU model		Yamashita model			CREED score
	Derivation	External validation	Derivation		External validation	Derivation and validation
Authors	Rodriguez R, et al.^15^	Chung-Esaki H, et al.^16^	Yamashita S, et al.^1^	Yamashita S, et al.^17^		Covino M, et al.^18^
Publication year	2011	2014	2021		2023	2023
Country	USA	USA	Japan		Japan	Italy
Study design	Triple-center, retrospective	Double-center, prospective	Single-center, case-control		Four-center, retrospective	Single-center, retrospective prospective
Setting	Emergency department		All departments			Emergency department
Objectives	Injection drug users admitted to hospital to exclude IE		Inpatients admitted to IE or undiagnosed fever			Inpatients due to fever
Number of IE patients	20 (9) / 236	18 (7) / 249	59 (41) / 144		119 (37) / 320	130 (1) / 13,163
Definition of IE	“Definite” of the modified DC^4^		“Definite” of the modified DC^4^			Not described
Age^§^	44 ± 11	42 ± 10	IE 71 (56-77) UF 66 (51-78)		69 (56-79)	70 (56-80)
Male (%)	154 (65)	159 (64)	72 (50)		177 (55)	7438 (56.5)
In-hospital death	11 (5)	10 (4)	15 (10)		26 (8)	1702 (12.9)
Number of predictive factors	3		5			12
AUC	0.670	0.800	0.893		0.783	Derivation 0.874, validation 0.881
Sensitivity	100	100	85		86	-
Specificity	-	13	85		64	-

Prediction Model for Intravenous Drug Users [15,16]

Two selected studies showed the results of the development and validation of an IE prediction model among intravenous drug users (IDUs) aged 17 years and older. In 2006, Rodriguez et al. conducted a retrospective observational study among IDUs aged 17 years and older. The study included patients admitted to the emergency departments of three medical institutions (in urban and rural areas) in the United States with a diagnosis of fever of unknown origin [15]. In total, 236 patients were included, with 20 cases of IE (8%). Participants meeting the “definite” criteria of the modified Duke criteria with a discharge diagnosis of IE were assigned to the IE group [4]. The model developed in this study used the presence or absence of skin infection, tachycardia, and heart murmur as predictive factors [15]. The model demonstrated a sensitivity of 100% and a negative predictive value of 100%. However, the area under the curve (AUC) was not assessed. Further limitations of this study included its retrospective nature, small sample size, and lack of validation.

To externally validate this model, Chung-Esaki et al. conducted a prospective observational study on febrile IDUs aged 17 years and older who were admitted to the emergency departments of two hospitals (one urban and one rural) in the United States for suspected endocarditis between June 2007 and March 2011 [16]. A total of 249 patients were included, with 18 cases of IE (7%). The inclusion criteria for the IE group were the same as those in the study by Rodriguez et al. [15]. The model was validated internally using the cohort from Rodriguez et al. and externally using the cohort from Chung-Esaki et al. The AUC was 0.670 for the internal validation cohort and 0.8 for the external validation cohort. The model demonstrated a sensitivity of 100%, a specificity of 13%, and a negative predictive value of 100%, with a post-test probability range of 3% to 20%. However, limitations of this study included the restriction to only two institutions with a small sample size and the reliance on patient self-reports to determine the presence or absence of intravenous drug use.

Prediction Model for Patients With Undiagnosed Fever or Admitted for IE [1,17]

Two selected studies showed the results of the development and external validation of an IE prediction model among patients aged 20 years and older who were admitted to all departments for IE or undiagnosed fever. Yamashita et al. conducted a single-center case-control study on IE cases admitted to a medical institution in Japan from 2007 to 2017 and undiagnosed fever cases admitted from 2015 to 2017 [17]. A total of 144 patients were enrolled, with 59 cases of IE (41%). IE was diagnosed according to the modified Duke criteria [4]. The predictive model developed in this study included the following: presence or absence of emergency transport, presence or absence of a heart murmur on admission, presence or absence of pleural effusion on admission, neutrophil percentage, and platelet count. The model demonstrated a sensitivity of 84.7%, a specificity of 84.7%, a positive predictive value of 55%, and a negative predictive value of 88%, with an AUC of 0.893 (95% confidence interval (CI): 0.828-0.959) and a shrinkage coefficient of 0.635. However, limitations of the study included its single-center design, a small number of IE cases, lack of external validation, and variability in the reasons for ambulance use across regions and countries.

To validate this model, Yamashita et al. conducted a retrospective observational study across four advanced acute care hospitals in urban, suburban, and rural areas in Japan [1]. The study included patients aged 20 years and older who were admitted for IE or undiagnosed fever between 2018 and 2020. A total of 320 patients were enrolled, including 119 cases of IE (37%). In this validation study, the model demonstrated a sensitivity of 86%, a specificity of 64%, a positive predictive value of 59%, and a negative predictive value of 88%, with an AUC of 0.783 (95% CI: 0.732-0.834) and a shrinkage coefficient of 0.963. A limitation of this study included the small number of participating institutions, despite being a multi-center study.

Prediction Model for Patients With Fever Admitted to the Emergency Department [18]

The selected study showed the results of the development and validation of an IE prediction model among patients with fever aged 18 years and older admitted to the emergency department. This study was conducted in two phases. The first phase was a retrospective observational study of patients with fever aged 18 years and older admitted to the emergency department of an urban medical institution in the United States from 2015 to 2019 [18]. The second phase was a prospective observational validation study of patients with fever admitted to the same emergency department from 2020 to 2021 [18]. The study included 15,689 patients in the derivation cohort and 13,163 patients in the validation cohort, with 267 (1.7%) and 130 (1.0%) cases of IE, respectively. Inclusion criteria for the IE group were not explicitly described in the text. The Clinical Rule for Infective Endocarditis in the Emergency Department (CREED) score developed in this study included 12 factors: sex (male), dialysis, chest pain, presence of a pacemaker, history of hospital discharge within one month, anemia, moderate to severe valvular disease, history of IE, stroke within one month, prosthetic valve, clinical symptoms suspicious for IE, and confirmed diagnosis of infection. The AUCs were 0.874 (95% CI: 0.849-0.899) in the derivation cohort and 0.881 (95% CI: 0.848-0.913) in the validation cohort. The coefficient of determination (R^2^), indicating model fit, was 0.332. Based on the model score, patients’ risk of IE was stratified into four groups: very low, low, high, and very high. In the derivation cohort, the positive predictive value and negative predictive value in the high-risk and very high-risk groups were 33.5% and 99.0% and 62.2% and 98.7%, respectively. In the validation cohort, they were 25.5% and 99.3% and 60.0% and 99.2%, respectively. Limitations of this study included its single-center nature, the relatively small number of IE cases compared to the total patient population, and the fact that approximately 70% of IE cases in the validation cohort fell into the low or very low-risk groups.

Discussion

IE remains a challenging disease to diagnose due to its diverse clinical manifestations, complex diagnostic criteria, and reliance on multiple advanced medical modalities for accurate diagnosis [1,6]. To assist physicians in timely and appropriately suspecting IE, a diagnostic prediction model that can be used shortly after a patient’s arrival rather than performing blood cultures is desired. The purpose of this study was to discuss the strengths and limitations of such existing models and to highlight the need for developing models that combine high accuracy and generalizability. As a result, three diagnostic prediction models were identified and analyzed in terms of their generalizability and accuracy.

The three models were developed in different countries: the United States, Japan, and Italy. Among these, the IE-IDU model developed by Rodriguez et al. and validated by Chung-Esaki et al. has the lowest generalizability as it predicts a diagnosis of IE in IDUs [15,16]. Although these studies were conducted in two to three medical institutions in both urban and rural areas in the United States, the participants were restricted to IDUs. While the proportion of IDUs among patients with IE is increasing in North America, it remains unchanged globally and has reportedly decreased to 6.8% in Europe and 2.2% in Japan [19,20]. Therefore, the target population to whom the model is applicable may vary among countries. In addition, the average patient age in the studies by Rodriguez et al. and Chung-Esaki et al. was in the 40s, influenced by the generally younger average age of patients with IE among IDUs, typically in their 30s [21]. Therefore, this model would be unsuitable for the growing population of older patients with IE seen in countries like Japan [20]. Furthermore, patients with IE patients were reportedly admitted to 16 different medical departments due to their diverse clinical manifestations [3]; however, the IE-IDU model would not be able to capture the full clinical picture of IE, because patients in the development and validation study of the model were restricted to IDUs admitted only to emergency departments. The CREED score, developed by Covino et al., is applicable to older febrile patients with IE [18]. However, as this was a single-center study of patients admitted to the emergency department, its generalizability is limited. The model developed by Yamashita et al. targets patients with IE and undiagnosed fever origin admitted to all medical departments and is suitable for febrile patients aged 20 years and older, including older ones. In addition, the model was validated in four medical institutions located in urban, suburban, and rural areas, indicating that the model has the highest generalizability among the three models. However, one factor in the model - ambulance use - may vary across countries or regions. The reasons for non-emergency ambulance use were reported as follows: reassurance gained from a physician’s advice about the patient's health problem; lack of knowledge and awareness of non-emergency visits; dissatisfaction with primary care providers; satisfaction with the quality of care provided in the emergency department and being able to receive more services at once; accessibility of the emergency department; recommendations for emergency department visits from healthcare professionals or non-medical professionals such as family and friends; and relationship between patients and medical providers (anonymity), such as being seen by a doctor they rarely see [22]. Therefore, it is uncertain whether similar results can be obtained in other countries or regions. Based on the above considerations, none of the three models currently demonstrate nationwide or global generalizability.

When evaluating the accuracy of prediction models, it is essential to consider their reliability. In developing prediction models, the number of events per variable should be at least 10 [23,24]. If it is less than 10, the model may overfit the specific dataset, potentially reducing its performance when applied to a different cohort. In addition, for the external validation of a prediction model, at least 100 cases in each of the event and non-event groups are required [25]. If the number of cases is less than 100, the model may overfit the specific dataset, leading to different results in another dataset [26]. Thus, a small sample size may cause a decrease in the reliability of the model, warranting discreet judgment when considering the reliability of the IE-IDU model. When a model includes a large number of factors, factors that are difficult to investigate at other hospitals may be included, potentially leading to overfitting. However, this has the advantage of enabling accurate risk stratification [27]. For instance, while the CREED score consists of as many as 12 factors, most of these factors can be derived from medical history, and the risk of overfitting may be low in institutions where echocardiography is available.

The AUC is a key indicator of the discriminative ability of the prediction models. AUC values of 0.6-0.7, 0.7-0.8, 0.8-0.9, and greater than 0.9 usually indicate poor or acceptable, fair, good, and excellent discrimination, respectively [28,29]. Among the three models reviewed, the CREED score had the highest AUC and the narrowest 95% CI in the validation cohort. Despite the low R^2^ value of 0.332, indicating high data variance, the high AUC suggests that the model captures important characteristics of IE. However, the CREED score is strongly influenced by the factor "suspicion of IE by the clinician," which may limit its reproducibility at other medical institutions or medical departments, resulting in underestimation of the possibility of IE in cases where suspicion is low. In addition, there was a significant dissociation between the number of IE and non-IE cases in the study by Covino et al. [18]. This may have biased the CREED score toward predicting non-IE cases, leading to an overestimated negative predictive value. A similar issue applies to the IE-IDU model. While the IE-IDU model demonstrated good discrimination with an AUC of 0.800 in the validation cohort, it had the lowest AUC of 0.670 in the development cohort among the three models. As noted above, the IE-IDU model may be affected by overfitting. By contrast, the model developed by Yamashita et al. exhibited lower AUC than the CREED score; however, it showed fair to good discrimination with a high shrinkage coefficient, suggesting that the model has high generalizability without overfitting to the data.

Limitations

This study has certain limitations. First, it is a narrative review based solely on a literature search conducted through PubMed rather than a systematic review. The present study may have a selection bias in the literature. Second, the reviewed studies were all observational and conducted in either a single or a limited number of facilities. Therefore, the obtained results may be affected by selection or information bias and some confounding factors. Nationwide or global validation of these models or randomized controlled trials to determine whether they can be widely used are warranted. Third, the understanding of one of the inclusion criteria, namely, “without using the blood culture results,” may not have been consistent between the two researchers who searched the literature. This might have led the Cohen's kappa being only 0.58, despite the 98.3% agreement rate, when two researchers initially discussed the results of the literature search. Both researchers rechecked the inclusion criteria, reaching complete agreement.

## Conclusions

Three diagnostic prediction models for IE are usable shortly after patient admission, regardless of whether the results of blood cultures have been reported. Among these, the model developed by Yamashita et al. demonstrates the highest generalizability, while the CREED score exhibits the best performance. However, none of these models possess nationwide or global generalizability, suggesting the necessity to collect data from a larger number of medical institutions and conduct high-level evidence studies such as randomized controlled trials.

## Appendices

Appendix 1: How to investigate PubMed using search terms

PubMed <January 1781 to October 2024>

1.    Infective endocarditis.tw. 47,305

2.    Infectious endocarditis.tw. 11,118

3.    1 or 2 = 47,305

4.    Prediction model 774,113

5.    Predictive model 774,113

6.    Prediction rule 17,282

7.    4 or 5 or 6 = 784,298

8.    3 and 7 = 405

Appendix 2: formula of each prediction model

IE-IDU Model (Developed by Rodriguez R et al. and Validated by Chung-Esaki H et al.)

Z = 0.63 [tachycardia] + 0.61 [cardiac murmur] - 1.0 [skin infection] −2.61

Yamashita Model (Developed and Validated by Yamashita S et al.)

Score = −8.350 + (transfer by ambulance = 4.087) + (cardiac murmur on admission = 2.844) + (pleural fluid = 1.440) + {neutrophil count (%) × 0.090} + {platelet count (103/µL) × (−0.057)}.

Predictive occurrence of infective endocarditis (%)

100 × exp(score) ÷ {1 + exp(score)}

CREED score (developed and validated by Covino M et al.)

Score = 1 [Male] + 1 [Dialysis] + 1 [Chest pain] + 1 [Pace maker] + 1 [Recent hospital discharge (< 1 mo)] + 2 [Anemia] + 2 [Moderate / Severe valvular disease] + 2 [Previous endocarditis diagnosis] +3 [Recent stroke (< 1 mo)] + 4 [Valvular prosthesis] + 11 [High Clinical suspicion] -2 [Ascertained infective diagnosis]
